# Treatment of colorectal leaks with the VACStent-Colon: results of the VACStent feasibility trial

**DOI:** 10.1007/s00464-026-13239-w

**Published:** 2026-08-04

**Authors:** Alexander Yohannes, Jonas Lange, Judith Knievel, Marion Brunner, Alois Fürst, Ulrich Hügle, Arno J. Dormann, Claus F. Eisenberger, Markus M. Heiss

**Affiliations:** 1 Department of Gastroenterology, Cologne-Holweide and Merheim Medical Center, Witten/Herdecke University, Ostmerheimer Str. 200, 51109 Cologne (Köln), Germany; 2 Department of Abdominal, Tumor, Transplant and Vascular Surgery, Cologne-Merheim Medical Center, Witten/Herdecke University, Cologne (Köln), Germany; 3 Surgical Department, Klinik Für Allgemein- Und Viszeralchirurgie, Caritas-Krankenhaus St. Josef, Regensburg, Germany

**Keywords:** VACStent, Colorectal leak, Anastomotic insufficiency, Endoscopic vacuum therapy, Intraluminal EVT, Stoma avoidance

## Abstract

**Background:**

Previously, the treatment of colorectal anastomotic insufficiencies with endoluminal vacuum therapy (EVT) was not feasible without creating a diverting stoma, as the suction sponge typically obstructed the intestinal lumen. However, clinical use of the VacStent GI™ in the upper gastrointestinal tract has demonstrated that secure closure of intestinal wall defects or anastomotic leaks, along with effective drainage of associated wound cavities, is achievable via intraluminal EVT. The innovative concept of the VacStent GI™ was now transferred to the lower GI tract.

**Method:**

A prospective multicenter feasibility study was conducted, with the primary endpoint being the safe and practical application of the device, complete coverage of the leak, and effective suction-based treatment of colorectal leaks. Secondary endpoints included the prevention of septic complications, avoidance of stoma creation, successful leak healing, and the assessment of adverse events such as stent migration, local erosion or ulceration, and bleeding.

**Results:**

Fourteen patients with postoperative colorectal anastomotic leaks and one with iatrogenic instrumental perforation were enrolled in two German hospitals. A total of 43 VacStent GI™ Colon were implanted, with a median of three VacStent per patient and a median treatment duration of 14 days. The safe and practical application of the VacStent GI™ Colon was achieved in all implantations, resulting in a leak healing rate of 93.3% (14/15 patients). There were no severe device-related adverse events (SADE) nor significant local bleeding or erosion. The creation of a diverting stoma was not necessary in any of the patients.

Minor stent-dislocation and migration, respectively, was observed in 4.7%. Three anastomotic stenoses were seen during follow-up.

**Conclusion:**

Clinical use of the VacStent GI™ Colon in the lower gastrointestinal tract demonstrates that anastomotic colonic leaks can be effectively treated, potentially avoiding the need for a diverting stoma.

Anastomotic leakages (AL) still present the major complication in colorectal surgery influencing perioperative outcome and even prognosis. Occurrence of AL is usually associated with an elevated mortality reaching up to 23% [1]. Patients suffering from AL generally need major surgical revision, usually leading to the creation of a diverting ostomy while the AL and its associated complications are being treated. Stoma reversal surgery performed after the healing of the typically inflamed anastomotic site and surrounding tissue inevitably necessitates often a multi-stage surgical approach. However, this is not always possible, e.g. in the case of a hostile abdomen or an unjustifiable perioperative risk.

After creation of a diverting ostomy, local therapy is usually required to facilitate closure of the AL [2]. Current medical standard consists of the local treatment via endoscopic vacuum therapy (EVT), to promote closure of a leakage and to drain extraluminal wound cavities. A sponge, usually made of open pore polyurethan, is inserted through the AL into the wound cavity. Negative pressure is established and maintained by an external vacuum pump to which the sponge is connected by a catheter that is routed out transanally. EVT has proven to be an efficient method of treating AL, with closure rates of around 80% [3]. There are a variety of other methods, including the usage of covered self-expanding metal stents (SEMS), endoscopic suturing, through the scope (TTS)- and over the scope (OTS)-clips as well as fibrin glue, all with varying success (= closure) rates [4–9]. They may present a viable alternative in specific circumstances but usually report inferior success rates compared to EVT which offers broader clinical applicability.

Since 2021, the VacStent GI™ (VACStent GmbH, Fulda, Germany) has become increasingly established in clinical practice within the upper gastrointestinal (GI) tract for the treatment of anastomotic leaks, particularly following esophagectomy [10]. The VacStent GI™ consists of a fully covered self-expanding metal stent (SEMS) constructed out of nitinol filaments. The VacStent GI™ Colon has a diameter of 24 mm built with an oral and aboral pulp and a total stent length of 80 mm.

The SEMS itself is encased in a 60-mm-long polyurethane sponge cylinder, which is connected to an embedded suction catheter. As is the case with conventional vacuum sponge systems, negative pressure is established through it by a continuously running vacuum pump. Prospective studies in the upper GI tract have shown closure rates comparative to those of sponge induced EVT while still maintaining free passage of fluids and mashed diet through the stent. The continuous suction prevented stent migration very effectively through the “suction cup” effect of the established negative pressure [10–13].

First clinical experience in the colorectal area confirmed that the VacStent GI™ can also be used in the lower GI tract and effectively supports wound healing [14]. However, free stool passage requires different dimensions for the VacStent GI™ [14]. This was taken into account in the design of the VacStent GI™ Colon with a stent diameter of 24 mm (Fig. 1). This initial prospective feasibility study was conducted to examine the clinical handling, the functionality and the potential complications of the VacStent GI™ Colon.

## Method and materials

### Investigational device and application

The VacStent GI™ Colon consists like its variation for the upper GI tract of a fully covered intestinal stent enclosed by a polyurethane sponge cylinder as described by Lange et al.[15] The application was performed as described before [14]. A continuous negative pressure of − 125 mmHg was applied as our institutional standard for the VacStent GI™ Colon, defined on the basis of our prior prospective experience with VacStent GI™ therapy in the esophagus.[15]. Lower pressures (− 75 to − 100 mmHg) were used at the discretion of the treating endoscopist on a case-by-case basis, in part because many procedures were performed in urgent, on-call settings.

### Study design

This multicenter, prospective single-arm study was designed to evaluate the feasibility and safety of treating lower gastrointestinal leaks using the VacStent GI™ Colon device that combines the principles of endoscopic vacuum therapy (EVT) and a fully covered nitinol SEMS. While three centers were initiated into this multicenter study, suitable patients were only included within two centers in Germany. The third initiated center did not enroll any patients, as no eligible cases presented during the study period. Procedures were carried out by experienced endoscopists at Cologne-Merheim Medical Center and Caritas-Krankenhaus St. Josef in Regensburg. The study was conducted in accordance with the ethical standards of the Declaration of Helsinki and received approval from the Institutional Review Board (IRB) of Witten/Herdecke University (Approval No. 124/2018). The trial was registered in the German Clinical Trials Registry (DRKS) under the identifier DRKS00016048.

### Trial endpoints

The primary objective of the study was to demonstrate the technical safety and feasibility of the VacStent GI™ Colon, specifically its ability to effectively seal colorectal leaks. The primary endpoint was considered fully achieved if the VacStent GI™ Colon could be successfully deployed, accurately positioned, and continuous negative pressure applied via the sponge-cylinder. This was verified endoscopically during the procedure and through ongoing monitoring of the applied vacuum pressure.

Secondary objectives included the evaluation of clinical signs of sepsis during the treatment course, assessment of the continuity of the stool passage, morphological healing of the leak, avoidance of a stoma, and the occurrence of any complications.

### Patient selection

Patients were eligible for inclusion if they had an endoscopically confirmed diagnosis of a lower GI leak—either postoperative (e.g. at an anastomotic site) or iatrogenic (resulting from endoscopic or surgical intervention)—and if the lesion could be reached using the VacStent GI™ applicator system. Informed consent was a prerequisite for participation.

Exclusion criteria included: leaks that could not be accessed endoscopically with the VacStent GI™ system, clinically unstable septic patients requiring immediate surgical interventions, patients with a full stomach and/or persistent vomiting with signs of clinical ileus, and individuals requiring full anticoagulation or presenting with thrombocytopenia (platelet count < 20.000/µl).

### Data collection and analysis

The safety, efficacy, and clinical course of VacStent GI™ Colon therapy were systematically monitored on a daily basis from patient enrollment through hospital discharge, and during scheduled follow-up visits up to 30 days post-discharge. All relevant data were recorded in standardized case report forms (CRFs), entered into a secure database, and analyzed. Morphological healing of the leak was assessed endoscopically at the end of VacStent GI™ Colon treatment and at the 30-day follow-up. Two patients who did not undergo follow-up endoscopy were assessed clinically by telephone. Contrast enema and cross-sectional imaging were not used routinely to confirm healing.

## Results

### Patient characteristics and performed procedures

In total, 15 patients in two German hospitals (13 in Cologne, 2 in Regensburg) had been enrolled in the trial between August 2023 and July 2025 (Table 1). The patients were 64 years old on average (34–92 years), 11 were male and 4 were female. The leaks were located at a median of 15 cm from the anal verge (range 8–20 cm; Table 1). Only one leak was located in the distal rectum (8 cm), whereas the remaining 14 were located between 10 and 20 cm, i.e. predominantly in the sigmoid colon and upper rectum.

**Table 1:** Patient, leak, and VacStent GI™ Colon treatment characteristics

Pat. No	Age, sex	ASA	disease	procedure	Localization of leakage (ab ano)	Days after surgery	Endoscopic treatment before stenting	VACStent, numbers	Duration of VACStent treatment, days	suction/ pressure (median), mmHg	SADE	clinical success	defecation
1	92, m	3	Colon descendens carcinoma	Left hemicolectomy with subtotal transverse colon resection	14 cm	14	None	2	11	−100	None	Yes	Stool conditioning
2	54, m	2	Chronic recurrent diverticulitis	Lap. sigmoid rectum resection en-bloc	8 cm	7	None	3	19	−125	None	Yes	Stool conditioning
3	64, m	3	Stenosing sigmoid tumor	Open sigmoid resection with bladder roof and small bowel segment	12 cm	4	None	3	1−6	−125	None	Yes	Stool conditioning
4	56, m	3	Rectum carcinoma	Laparoscopic anterior rectal resection	11 cm	2	None	5	34	−125	None	Yes	Stool conditioning
5	58, m	3	Chronic fistula at the height of the descendorectostomy	Laparoscopic Hartmann's reversal	18 cm	132	None	2	14	−125	None	No	Persistent diarrhoea
6	67, w	3	Colectomy for colonic ischemia	Stoma reversal	18 cm	6	None	2	8	−125	None	Yes	Stool conditioning
7	49, m	2	Sigma carcinoma	Sigmoid resection	15 cm	11	None	3	21	−125	None	Yes	Stool conditioning
8	77, m	3	Sigma carcinoma	Sigmoid resection	15 cm	7	None	3	14	−125	None	Yes	Stool conditioning
9	59, w	3	Iatrogenic rectal wall perforation	Transmural defect after usage of a stool collector during ICU treatment	12 cm	iatrogenic	None	2	12	−100	None	Yes	Stool conditioning
10	63, m	2	Descendostoma after sigmoid perforation, sigmoid diverticulitis	Stoma reversal	15 cm	8	None	2	9	−125	None	Yes	Stool conditioning
11	34, m	3	Sigmoid diverticulitis	Sigmoid resection for perforated sigmoid diverticulitis with peritonitis	20 cm	3	None	3	18	−100	None	Yes	Stool conditioning
12	79, m	3	Stenosing, bleeding adenocarcinoma of the sigmoid colon	Lap. Sigmoid resection	20 cm	6	None	5	29	−125	None	Yes	Stool conditioning
13	64, w	3	Carcinoma of the ascending colon with suspected secondary carcinoma of the sigmoid colon	Robot−assisted right hemicolectomy and anterior rectal resection	12 cm	8	None	2	14	−100	None	Yes	Stool conditioning
14	59, m	1	Sigmoid diverticulitis with abscess	Lap. Sigmoid resection	15 cm	15	None	4	16	−125	None	Yes	Stool conditioning
15	85, w	3	Sigmoid diverticulitis, rectal stenosis, outlet obstruction syndrome	Lap. Anterior rectum resection	10 cm	4	None	2	14	−75	None	Yes	Stool conditioning

Seven patients were diagnosed with colorectal cancer, four with sigmoid diverticulitis. These eleven patients developed an anastomotic leakage 2 to 15 days after colorectal resection.

One patient had a colectomy with creation of a terminal ileostomy because of colon ischemia (Hartmann-procedure). After two years, the surgery with stoma reversal and ileorectostomy was performed. The leakage occurred 6 days after creation of the anastomosis.

One patient underwent a Hartmann’s sigmoid resection with creation of an end colostomy due to intestinal gangrene with colon perforation. About five months later, the stoma was resected and an anastomosis applied. In the meantime, the patient received immunotherapy following the diagnosis of metastatic renal cell carcinoma. Due to symptomatic hyponatremia, the patient was admitted to the hospital by our colleagues from the oncology department. During the inpatient stay, rising inflammatory laboratory parameters led to a computed tomography (CT) of the abdomen, which revealed a leakage at the height of the anastomosis. The finding was correlated endoscopically. Given that the leakage was detected on postoperative day 132, we considered it an iatrogenic chronic fistula at the height of the anastomosis following sigmoid resection. We therefore decided to proceed with the implantation of a VacStent GI™ Colon.

One patient’s rectum wall perforated following the removal of a stool management catheter, which had been inserted several days before.

One patient developed anastomotic leakage after reconnection surgery for an end descending colostomy. The stoma was created after sigmoid perforation due to diverticulitis.

### VacStent GI™ Colon treatment

In 15 patients diagnosed with a colorectal leak in total 43 VacStent GI™ Colon were placed endoscopically (Fig. 2). In all patients the VacStent GI™ Colon was applied as the primary endoscopic treatment, without preceding sponge-based EVT (Table 1). In all cases correct positioning and deployment of the VacStent GI™ Colon was technically successful. Placement was reported to be easy (97.7%), only in one case difficult (Table 2). The leak was covered completely, and in all cases continuous suction with a median suction of––125 mmHg (range − 75 to − 125 mmHg) was installed. The primary endpoint was met in all patients (Table 3).

**Table 2:** Results: VACStent applicability

	*N* (%)
Patients treated (*n*)	15 (100)
VACStent placements	43 (100)
Median no. VACStents/patient	3
Median total hospital stay (days)	19
Ease of placement	
Easy	42 (97.7)
Moderate	0 (0)
Difficult	1 (2.3)
Impossible	0 (0)
Leak coverage	
Easy	43 (100)
Moderate	0 (0)
Difficult	0 (0)
Impossible	0 (0)
Establishment of negative pressure	
Easy	43 (100)
Moderate	0 (0)
Difficult	0 (0)
Impossible	0 (0)
VACStent removal	
Easy	41 (95.3)
Moderate	0 (0)
Difficult	0 (0)
Impossible	2 (4.7) *

^*^ VACStent was removed by the patients themselves

**Table 3:** Results: Study endpoints

Items	*N* (%)
Primary endpoint	
Technical success/leak coverage/suction application	43 procedures (100)
Secondary endpoints	
Complete closure of leak	14/15 (93.3)
Stoma creation	0/15
Clinical signs of sepsis	0/15
Anastomotic stenosis	3/15
Impaired stool passage	0/15
Mortality	0/15

Complete morphological healing of the leak, confirmed endoscopically, at the end of the VacStent GI™ Colon treatment was seen in 12 out of 15 patients (80%). In two of the three remaining patients the residual minimal leak closed spontaneously and uneventfully within four weeks after the VacStent GI™ Colon treatment, yielding an overall leak closure of 14 out of 15 patients (93.3%). One patient (Table 1, no. 5) did not achieve leak closure.

The median VacStent GI™ Colon treatment time was 14 days (range 8–34), the median indwelling time for the VacStent GI™ Colon was 7 days (range 1–8). The median number of VacStent GI™ Colon applications per patient was 3 (range 2–5).

All patients were able to avoid a diverting stoma. Stool conditioning was performed by Macrogol.

3 bags/day in 14 patients, and fiber-free nutrition was advised to ensure a soft fecal passage. One patient did not receive Macrogol. This patient had persistent diarrhoea as part of their immunotherapy for renal cell carcinoma.

In no case was a severe adverse device associated event (SADE) reported (Table 4). In particular, VacStent GI™ Colon migration or dislocation was observed in two cases. These two stents were pulled out by the patients themselves while delirious (after one day) or accidentally at night (after two days).

**Table 4:** Results: VACStent complications

	*N* (%)
Dislocation/migration	
None	41 (95.3)
Rarely	0 (0)
Significant	0 (0)
Massive	2 (4.7)
Erosion/ulcer	
None	34 (79.1)
Rarely	8 (18.6)
Significant	1 (2.3)
Massive	0 (0)
Local bleeding	
No	38 (88.4)
Rarely	5 (11.6)
Significant	0 (0)
Massive	0 (0)

Moreover, one clinically significant severe ulcer was noted (2.3%) and also 5 (11.6%) rarely local bleeding. No significant malfunctions or clogging of the drainage capacity of the VacStent GI™ Colon were reported.

Removal of the 43 VacStent GI™ Colon was without major problems. For two VacStent GI^TM^ Colon the technical removal was not possible because these were already pulled out by the patients themselves.

Follow-up 30 days after patient discharge was done endoscopically in 13 of the 15 patients. Two patients were contacted by phone presenting uneventfully. Three patients developed stenosis, which was treated endoscopically with balloon dilatation. One of the three patients underwent anastomotic resection eight months later. One other patient underwent balloon dilatation after six months, which led to perforation, requiring surgery.

In all other patients no clinically significant complications were observed.

## Discussion

It is evident that effective treatment of AL still requires extensive research efforts. VacStent GI™ Colon presents a very promising new alternative in the toolset of an endoscopist in treatment of AL. A technical success rate of stent deployment was reached in every case with only one VacStent GI™ Colon application described as difficult while every other placement of 42 VacStent GI™ Colon was described as easy, indicating that the implantation is an easy to master task for experienced endoscopists. Functional patency which allows adequate stool passage of the stent lumen was ensured in every case in which the VacStent GI™ Colon was used, confirming the basic functionality of the hybrid system even under the physiological conditions of the colon and the pathophysiological conditions of the operated colon. The safety profile is particularly noteworthy: there were no SADEs such as iatrogenic perforations or clinically relevant bleeding. In our opinion, this data underlines that the VacStent GI™ Colon is safe to use in case of AL.

Evaluating the clinical value of the VacStent GI™ Colon, a comparison with established standards is necessary. Classic EVT offers good wound debridement and wound conditioning, but inevitably leads to an occlusion of the intestinal lumen, impeding physiological passage of stool [16, 17 ]. SEMS on the other hand warrant lumen patency, but suffer under oftentimes intolerably high migration rates of up to 40% [18]. The reason lies within its configuration: While there is usually no mucosal ingrowth to be detected, SEMS usually are not fixed to the intestinal wall. VacStent GI™ Colon seems to solve this obstacle with its synergistic approach. It combines the active drainage of the EVT with lumen patency of a SEMS. The decisive mechanism of action here seems to be the physical interaction of the negative pressure with the intestinal wall. The vacuum sucks the sponge-coated stent onto the mucosa, creating a “suction cup effect”, which seems to almost completely eliminate stent migration compared to conventional SEMS and enables stable anchoring during peristalsis. Latter needs to be considered a much higher obstacle in the lower GI tract than in the upper GI tract again emphasizing that the VacStent GI™ Colon might prove to be a suitable alternative for treating AL.

**Fig. 1:**
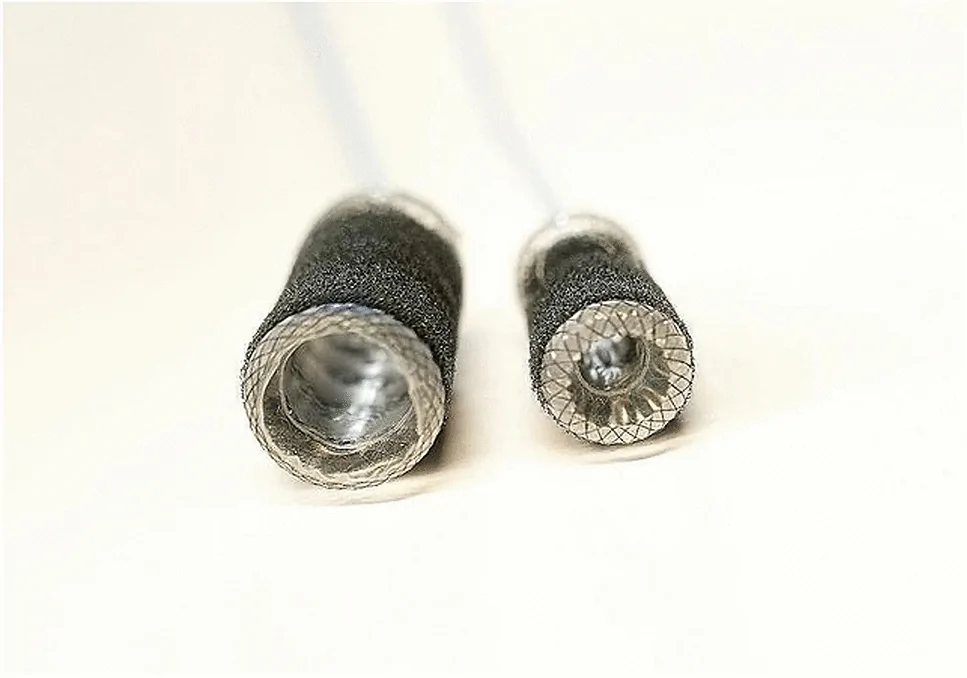
Side-by-side comparison of the VacStent GI™ Colon (left) with the already clinically established VacStent GI™ (right), which is used for leakages following upper GI surgery. The VacStent GI™ Colon has a larger stent diameter of 24 mm compared to the smaller VacStent GI™ with a diameter of 14 mm

In our previously published experiences with the usage of the VacStent GI™ as a therapeutic device for treating and prophylactic device for preventing AL in colorectal surgery, we reported two stent dislocations [14]. Those dislocations happened when using the smaller VacStent GI™ developed for the upper GI tract. In our feasibility study we observed two dislocations, which were accidental removals of the device by patients themselves with no dislocation being observed due to insufficient colonic wall adherence or inappropriate suction pressure.

During the conduct of this study, Ramser et al. reported a retrospective single-center experience with the same VacStent GI™ Colon device in a comparable cohort [19]. Thirteen patients received a total of 47 VacStent GI™ Colon, with continuous luminal patency achieved in 89.4%, closely mirroring the uninterrupted stool passage observed in our series. These findings corroborate the feasibility and safety we report and provide complementary evidence, given their retrospective and our prospective, multicenter design. Two aspects are worth noting here. First, in their therapeutic subgroup most patients had a diverting loop ileostomy in place or subsequently received one, and only a single patient was managed without a stoma, whereas no patient in our cohort required a stoma. This difference may partly reflect the more proximal leak localisation in our series (median 15 cm from the anal verge). Second, Ramser et al. identified pain as the most frequent complication, particularly with distally placed stents, which is consistent with the predominantly proximal localisation and the low rate of clinically relevant local complications in our cohort. Finally, they additionally explored prophylactic VacStent GI™ Colon placement in high-risk anastomoses without a diverting stoma, with no subsequent leakage which certainly seems to be a promising direction that warrants prospective evaluation.

**Fig. 2:**
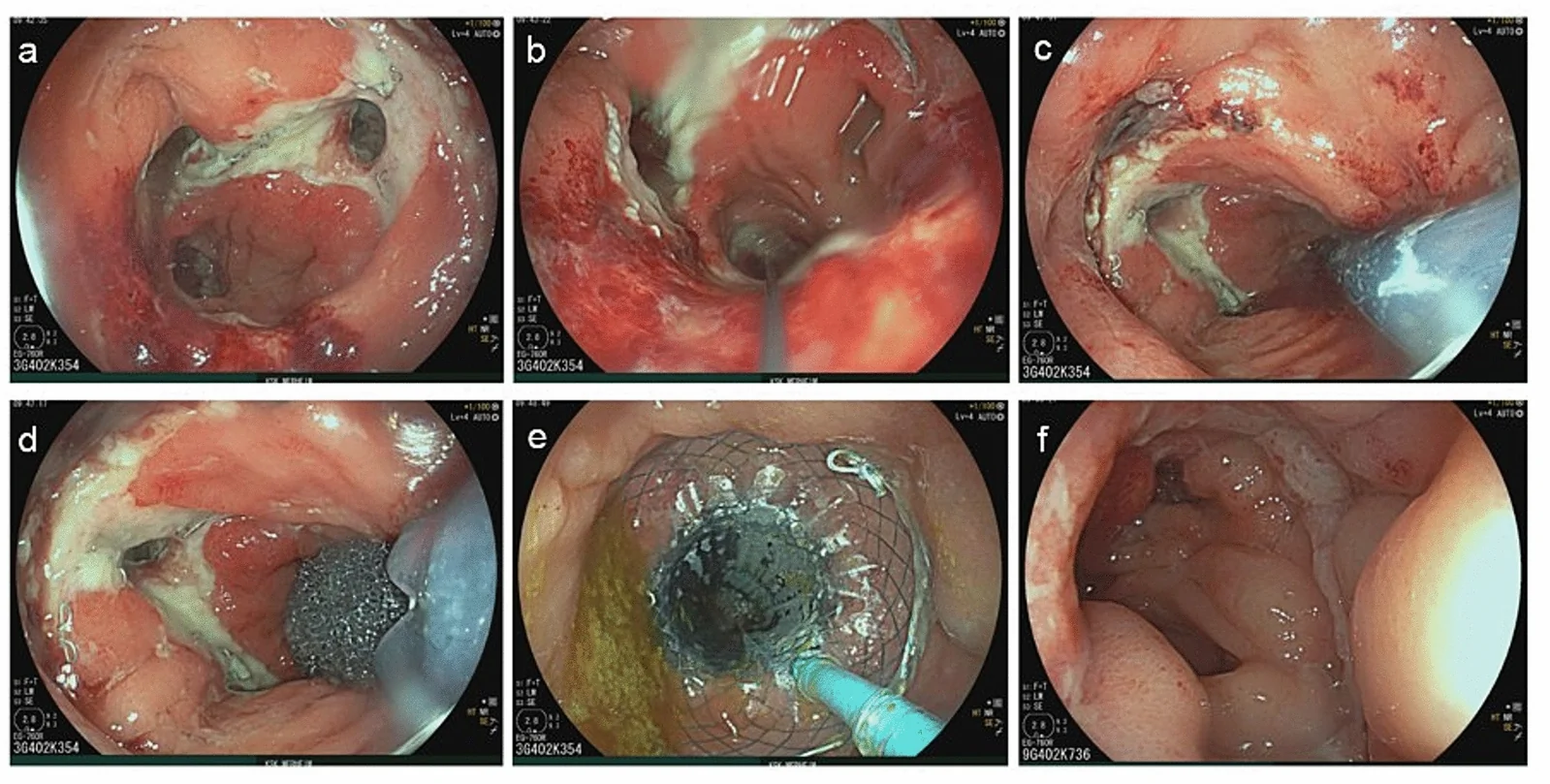
Exemplary insertion of a VacStent GI™ Colon following a large anastomotic insufficiency after total anterior rectal resection (**a**) with an extraluminal wound cavity of around 5 × 5 cm. A guide wire is placed (**b**), through which the application device holding the stent is led to the site of the anastomotic leakage (**c**). The VacStent GI™ Colon is slowly released, beginning with the oral pulp that is placed around 2 cm orally of the leakage (**d**). After the stent is completely released, adequate negative pressure is established to facilitate debridement and stability (**e**). After the complete course of treatment with the VacStent GI™ Colon is finished, the anastomotic leakage has been resolved (**f**)

Limitations of this study need to be addressed. The small study cohort of 15 patients with 43 VacStent GI™ Colon applications without a control group prevent this study from evaluating a potential advantage of using the VacStent GI™ Colon instead of classic EVT combined with a necessary surgical stoma application. The cohort is clinically heterogeneous since it includes patients getting AL after surgery following malign and benign conditions as well as an iatrogenic lesion. There was also no predefined regimen on always using a certain suction pressure. While in most cases, a suction pressure of −125 mmHg was documented, there have been variations starting from only −75 mmHg. A reliable statement of suction pressures and their effect on the resolution of AL is therefore not possible. Nor is it possible to determine the minimum negative pressure at which AL resolution is still possible. The optimal level of negative pressure for colorectal application has not been established. Based on experience with polyurethane sponge systems and with the VacStent GI™, higher negative pressures above 100 mmHg are thought to enhance the buildup of granulation tissue and to enhance the “suction-cup effect”, thereby further counteracting stent migration, although robust objective data are lacking and this remains a matter of expert opinion. On the other hand, an excessively high negative pressure might, under certain anatomical conditions, contribute to erosion of adjacent tissue. In the absence of comparative data, the level of negative pressure was therefore individualised by the treating endoscopist. Patient comfort and device tolerability were not systematically assessed with a validated patient-reported outcome instrument. That said, no patient discontinued therapy because of discomfort. Given the pain associated with distally placed stents reported in the retrospective series discussed above, systematic assessment of patient comfort should be included in future studies.

In the context of said limitations, the documented complications appear overall to be manageable without any SADE with a high degree of feasibility. Technical success with leak coverage and establishment of a negative pressure is reported for all 43 applications, placement was almost always described by endoscopists as “easy” (42/43), and removal was also mostly uncomplicated (41/43), with two impossible removals explained by patients removing the stent themselves. This is consistent with the fact that migration and displacement is rarely reported overall (2/43 as "massive"), meaning that a VacStent – migration and dislodgement is only a minor problem in this data set.

Mucosal lesions appear more frequently but are mostly mild and without clinical significance. Erosions or ulcers are predominantly not seen at all (34/43), otherwise mostly documented as “rare” (8/43) and only once as “significant.” Relevant bleeding is not reported, “rare,” meaning limited, bleeding occurred in 5/43 applications.

The most notable late complication in the endpoints is anastomotic stenosis (20 percent). Without claiming statistical significance in the context of this feasibility study, it should be emphasised that the three patients who experienced anastomotic stenosis later on required treatment with more than two consecutive VacStent GI™ Colon probably indicating to have larger and more complex AL.

The advantage for patients lies primarily in the sealing of the AL combined with the patency of the stent for stool passage, which can replace surgical revision and stoma creation. The preserved intestinal passage allows for an evacuation of gas buildup and liquified stool via naturalis and has the potential to significantly increase patient comfort compared to total obstruction caused by an intraluminal sponge alone. As part of our feasibility study, patients received stool conditioning with macrogol, which enabled us to achieve mushy, liquid stool consistency. Patients did not need to follow a specific and strict nutritional regimen solely aimed at preventing stent obstruction. Patients were instructed to eat a diet mainly consisting of calorie dense fiber-free liquid food. Some patients ate fiber-free soft food as well. Fortunately, no stent occlusion was observed at any time during stool conditioning with macrogol. In initial experiences with VacStent GI™ in the esophagus, stent occlusions were described by Chon et al., which Lange et al. were unable to reproduce in a larger prospective multicenter feasibility study [10–12]. In our study, no patient showed clinical signs of intestinal obstruction, nor did the control endoscopies ever indicate existing or impending stent occlusion.

On the other hand, no patient required a diverting ostomy after the identification of AL, and leak closure was achieved in 14 of 15 patients (93.3%) under treatment with a VacStent GI™ Colon. In our opinion, these results are indicative of a safe usage of the VacStent GI™ Colon in cases in which an AL is detected, and an immediate rescue surgery is not necessary. Further studies need to verify these first experiences we have gathered regarding the preventability of a stoma. In addition to the evident advantages for patients of potentially avoiding a stoma, also the economic argument must be taken into account. In particular, the high follow-up costs of stoma care and the need for a second surgery for stoma resolution should be mentioned here [20]. At the same time, the acquisition cost of the device itself must be considered: with a median of three VacStent GI™ Colon per patient, the cumulative device cost is not negligible. As list prices vary considerably between countries and institutions, a single generalizable figure cannot be provided. A formal cost-effectiveness analysis weighing device expenditure against the avoided costs of stoma creation, stoma care, and reversal surgery was beyond the scope of this feasibility study and should be addressed in future work. Based on the study data, it can be expected that the patient’s quality of life will be significantly improved by the use of the VacStent GI™ Colon.

## Conclusions

VacStent GI™ Colon proves to be a new feasible therapy option for the management of AL. Through its hybrid approach, already established in the upper GI tract, it aims to combine the advantages of active wound drainage and debridement of EVT with lumen patency for stool passage seen in SEMS. This demonstrates its potential in substituting for deviating stoma in AL treatment.
